# How to Start Up a National Wildlife Health Surveillance Programme

**DOI:** 10.3390/ani11092543

**Published:** 2021-08-30

**Authors:** Becki Lawson, Aleksija Neimanis, Antonio Lavazza, Jorge Ramón López-Olvera, Paul Tavernier, Charalambos Billinis, James Paul Duff, Daniel T. Mladenov, Jolianne M. Rijks, Sara Savić, Gudrun Wibbelt, Marie-Pierre Ryser-Degiorgis, Thijs Kuiken

**Affiliations:** 1 Institute of Zoology, Zoological Society of London, Regent’s Park, London NW1 4RY, UK; 2 Department of Pathology and Wildlife Diseases, National Veterinary Institute (SVA), 751 89 Uppsala, Sweden; aleksija.neimane@sva.se; 3 Istituto Zooprofilattico Sperimentale della Lombardia e dell’Emilia Romagna, via Bianchi 7/9, 25124 Brescia, Italy; antonio.lavazza@izsler.it; 4 Wildlife Ecology & Health Group and Servei d’Ecopatologia de Fauna Salvatge, Departament de Medicina i Cirugia Animals, Universitat Autònoma de Barcelona, Travessera dels Turons s/n, Bellaterra, 08193 Barcelona, Spain; Jordi.Lopez.Olvera@uab.cat; 5 WILDPAD, Polbroek 17, 9520 St-Lievens-Houtem, Belgium; paul_tavernier@skynet.be; 6 Department of Microbiology and Parasitology, Faculty of Veterinary Science, Trikalon-Str. 224, 43100 Karditsa, Greece; billinis@vet.uth.gr; 7 Animal and Plant Health Agency, Diseases of Wildlife Scheme, Penrith CA11 9RR, Cumbria, UK; Paul.Duff@apha.gov.uk; 8 KRKA Bulgaria EOOD, 1000 Sofia, Bulgaria; dr.danmladenov@gmail.com; 9 National Diagnostic Research Veterinary Medical Institute “Prof. Dr. Georgi Pavlov”, 1000 Sofia, Bulgaria; 10 Dutch Wildlife Health Centre (DWHC), Utrecht University, Yalelaan 1, 3584 CL Utrecht, The Netherlands; J.M.Rijks@uu.nl; 11 Scientific Veterinary Institute “Novi Sad”, Rumenacki put 20, 21000 Novi Sad, Serbia; sara@niv.ns.ac.rs; 12 Leibniz Institute for Zoo and Wildlife Research, 10315 Berlin, Germany; wibbelt@izw-berlin.de; 13 Institute for Fish and Wildlife Health, Vetsuisse Faculty, University of Bern, Postfach, Länggass-Str. 122, 3001 Bern, Switzerland; marie-pierre.ryser@vetsuisse.unibe.ch; 14 World Organisation for Animal Health (OIE) Working Group on Wildlife, 12 rue de Prony, 75017 Paris, France; 15 Department of Viroscience, Erasmus University Medical Centre, 3015 GD Rotterdam, The Netherlands; t.kuiken@erasmusmc.nl

**Keywords:** disease, general, scanning, surveillance, targeted, partnership, network, wildlife, health

## Abstract

**Simple Summary:**

A sound understanding of wildlife health is required to inform disease management and mitigation measures in order to help safeguard public, livestock, companion animal and wildlife health. Whilst multiple countries in Europe have schemes for wildlife health surveillance (WHS) in place that monitor the disease conditions that affect free-living wildlife, these vary in scope and scale. In 2018, the Network for WHS of the European Wildlife Disease Association hosted a meeting where representatives from countries with variable levels of current WHS were invited to share knowledge and experience of how their programmes began or were expanded. Through a series of presentations, the events that led to the start-up and expansion of WHS programmes were highlighted, such as the creation of action plans and collaboration through partnership formation. Challenges to development were identified, including limited funding and logistical difficulties around data sharing and the harmonisation of methods. Following a panel discussion, a series of practical recommendations were formulated, offering guidance on how to overcome key challenges for the instigation of national WHS programmes. It is hoped that this resource will provide a useful tool to help support the creation and expansion of WHS programmes in Europe and beyond.

**Abstract:**

Whilst multiple countries in Europe have wildlife health surveillance (WHS) programmes, they vary in scope. In many countries, coordinated general surveillance at a national scale is not conducted and the knowledge of wildlife health status in Europe remains limited. Learning lessons from countries with established systems may help others to effectively implement WHS schemes. In order to facilitate information exchange, the WHS Network of the European Wildlife Disease Association organised a workshop to both collate knowledge and experience from countries that had started or expanded WHS programmes and to translate this information into practical recommendations. Presentations were given by invited representatives of European countries with different WHS levels. Events that led to the start-up and fostered growth spurts of WHS were highlighted, including action plan creation, partnership formation, organisation restructuring and appraisal by external audit. Challenges to programme development, such as a lack of funding, data sharing, infrastructural provision and method harmonisation, were explored. Recommendations to help overcome key challenges were summarised as: understanding and awareness; cross-sectoral scope; national-scale collaboration; harmonisation of methods; government support; academic support; other funding support; staff expertise and capacity; leadership, feedback and engagement; and threat mitigation and wildlife disease management. This resource may enable the development of WHS programmes in Europe and beyond.

## 1. Introduction

Wildlife health surveillance (WHS) in Europe is fragmented and incomplete. In the most recent survey on WHS programmes carried out in 2009, only 14 of the 25 European countries that participated had established partial or comprehensive general WHS [1]. This suggests that knowledge of the status of wildlife health is limited in the majority of European countries. 

A sound understanding of wildlife health is required to inform disease management and mitigation measures in order to help safeguard public, livestock, companion animal and wildlife health. Free-living wildlife can act as sources of zoonotic pathogens and reservoirs of agents with significant economic implications for livestock trade. Diseases are increasingly recognised as a threat to biodiversity, with anthropogenic drivers of emergence including pathogen pollution through host and agent translocation [2,3]. Wildlife health surveillance can also form part of integrated programmes to appraise wider ecosystem health; for example, through the use of sentinel free-living species in order to assess contaminant exposure. 

### 1.1. Historical Overview of WHS in Europe 

As part of the inaugural EWDA Network meeting in 2009, an overview of the status of WHS in Europe was obtained by a questionnaire survey among representatives from 25/49 European countries [1]. For this survey, WHS was divided into general surveillance and targeted surveillance (Table 1). The survey participants were asked to categorise their country’s surveillance level based on the following classification: level 1: no general surveillance; level 2: partial general surveillance; level 3: comprehensive general surveillance (Table 1). Out of the 25 participating countries, eleven had surveillance level 1, eight had level 2, and six had level 3. Instead of, or in addition to, general wildlife health surveillance, several countries performed targeted surveillance. Whilst 16 diseases were cited as the focus of targeted programmes, the most frequent examples in descending rank order comprised rabies, avian influenza, tuberculosis, classical swine fever and trichinellosis.

The intensity of surveillance and funding sources varied greatly per country, with the number of animals examined post-mortem ranging from 30 to 5000 per year for general surveillance programmes, and from tens to tens of thousands per year for targeted surveillance programmes. By far the most important funding for WHS was provided by national governments, with additional funding from hunter organisations, universities, research projects, non-governmental organisations, the agricultural industry and environmental organisations.

### 1.2. Requirements of a WHS Programme

The World Organisation for Animal Health (OIE) recommends that every country has a set of government policies, regulations and programmes to effectively manage issues related to pathogens in wildlife, because countries that are not prepared are at increased risk of experiencing significant impacts from wildlife-related diseases [4]. Furthermore, in response to global trends in disease emergence and biodiversity loss, there is recognition of an urgent need to strengthen the wildlife component of One Health [8]. To this end, the OIE has developed the document ‘Wildlife Health Framework: Protecting wildlife health to achieve One Health’ [9].

National wildlife disease surveillance programmes generally have two primary objectives: (1) to reduce the social, human health, economic and ecological costs of pathogens carried by wild animals; and (2) to meet international obligations to detect and report important pathogens occurring in wild animals [4]. Essential components of a national wildlife disease/health programme comprise: prevention; early detection (surveillance); timely decisions and responses; and effective pathogen management [4].

The core purposes of national wildlife health programmes should include “establishment and communication of the national wildlife health status; leading national planning; centralising information and expertise; developing national networks leading to harmonisation and collaborations; developing wildlife health workforces; and centralising administration and management of national programmes” [10].

Surveillance, defined as “the systematic on-going collection, collation, and analysis of information related to animal health and the timely dissemination of information to those who need to know so that action can be taken” [4] is an essential component of health programmes. The OIE advises that a WHS programme comprises several core components, including the detection and identification of diseases/pathogens; analysis and communication (requiring input from epidemiologists, wildlife biologists and ecologists); and information management (collection of case/sample metadata, observation of wildlife mortality or sickness and data submission to notification systems) [11]. Depending on the needs, objectives and resources, different types of surveillance may be carried out: general or targeted (Table 1). Targeted surveillance activities can follow a risk-based design (focused on areas with the highest probability of occurrence or with the most serious expected consequences) or be part of an adaptive strategy (complementing general surveillance activities as needed). Surveillance may rely not only on laboratory diagnostics but also on participatory approaches (stakeholder knowledge, citizen science) and/or syndromic approaches (identifying case clusters with common characteristics before an aetiological diagnosis is made) [12,13,14]. In order to be effective and comprehensive, a surveillance programme should investigate the occurrence and epidemiology of infection and disease in free-living wildlife through approaches that include general and targeted surveillance, outbreak investigation and the archiving of biological samples [4,5,11,13].

In practice, a WHS programme requires a network of field partners submitting material to diagnostic laboratories with expertise in wildlife diseases, the analysis and storage of diagnostic samples and data and the communication of results to stakeholders. Depending on the country, a national wildlife health programme may rely on a network of local surveillance programmes (i.e., decentralised) or on a single countrywide programme (i.e., centralised) [10].

Starting a WHS programme is challenging. Knowing how other European countries with a WHS programme began would be helpful, but such information is not generally available. Therefore, the Network for WHS of the European Wildlife Disease Association (hereafter EWDA Network) (http://ewda.org/ewda-network/, accessed on 21 June 2021) organised a workshop with the goal to bring together knowledge on WHS and first-hand experience from people who had started up or expanded WHS programmes in their countries. Another objective was to translate this information into practical recommendations, as an aid for countries to set up their own WHS programmes.

## 2. Materials and Methods

The workshop was held in Larissa, Thessaly, Greece on 26 August 2018, and attended by 57 people from 21 countries. After introducing the main concepts about WHS and its history in Europe, the start-ups and growth spurts of WHS programmes of six countries were presented. These presentations were complemented by situation reports from four countries lacking a WHS programme, which outlined challenges to programme development. Detailed country situation reports are available in the workshop report (https://ewda.org/ewda-network-reports/, accessed on 31 July 2021). These presentations were followed by panel discussions about what had worked and what had not and the most important recommendations to overcome challenges and start up a WHS programme.

## 3. Results

### 3.1. Start-Up and Growth Spurts of Established WHS Programmes

Six European countries with WHS programmes at level 2 or 3, comprising Belgium, Great Britain, Italy, the Netherlands, Spain and Switzerland, provided insight into programme establishment and development. These programmes incorporate varying degrees of general and targeted surveillance, in large part reflecting the initial steps taken towards programme formation. National WHS programmes in Switzerland, Great Britain and the Netherlands, and the regional programme in Lombardy, Italy, were set up as general surveillance programmes. They are founded on the investigation of morbidity and mortality events in wildlife for early disease detection, alone or together with targeted surveillance, and guide disease management actions. In Switzerland, the programme began in 1962 as a small, university-based pathology unit and has evolved into a more comprehensive national programme rooted in wildlife disease ecology (Institute for Fish and Wildlife Health, https://www.fiwi.vetsuisse.unibe.ch/, accessed on 21 June 2021) [15]. In Great Britain, the programme originated from a governmental general surveillance scheme (Diseases of Wildlife Scheme, http://apha.defra.gov.uk/vet-gateway/surveillance/seg/wildlife.htm, accessed on 21 June 2021) set up in England and Wales in 1998 in parallel with new livestock surveillance schemes. In 2009, the Great Britain Wildlife Disease Surveillance Partnership (GBWDSP) was formed to conduct national WHS under the leadership of the Animal and Plant Health Agency. In the Netherlands, the Dutch Wildlife Health Centre (https://www.dwhc.nl/, accessed on 21 June 2021) was established in 2002 to fill the void of general WHS and to coordinate pre-existing wildlife health initiatives, including the international reporting of wildlife diseases. In the Lombardy region, Italy, the programme was established in 2012 to coordinate pre-existing, but isolated, monitoring activities in order to gain a more complete picture of the regional status of wildlife diseases [16]. This programme is similar to the one in force in the neighbouring Emilia-Romagna region since 2006 [17].

In contrast, WHS programmes in Spain and Belgium were founded upon, and are delivered through, predominantly targeted surveillance. In Spain, WHS began in the 1990s as smaller, regional initiatives of primarily targeted surveillance. In 2003, the *Plan Nacional de Vigilancia Sanitaria en Fauna Silvestre* (PVSFS) established the Spanish national WHS programme, integrated in a series of protocols to monitor and manage wildlife diseases [18]. This exemplifies how the coordination and harmonisation of smaller, regional initiatives can result in a national WHS scheme. This targeted surveillance information is integrated with general surveillance information, most of which is generated through rehabilitation centres, nature management organisations, hunting associations, universities and research institutions, collected first at a regional scale, and then coordinated at a national level. For this purpose, standardised protocols and actions in the case of finding diseased or dead wildlife are outlined by the central administration and disseminated to the regional administrations, stakeholders and the public [19].

In Belgium, in Wallonia, the academic and regional governmental partnership (*Réseau de Surveillance Sanitaire en Faune Sauvage*, http://www.faunesauvage.be/, accessed on 21 June 2021) arose from targeted pathogen surveillance in game species. Simultaneously, the Belgian Wildlife Disease Society (https://bwds.be/, accessed on 21 June 2021) became established in 2004 and played a key role in facilitating the international reporting of wildlife diseases. Subsequently, WHS started officially in Flanders too, and the federal government now receives data for the international reporting of wildlife diseases from the regional governments.

Existing programme activities that are mandated by authorities are funded, at least in part, by the government. However, the delivery varies widely, illustrating flexibility in how working practice can be adapted to reflect the situation. For example, the Belgian programme in Flanders is funded and delivered almost exclusively by governmental authorities. Whilst regional authorities are also responsible for programme delivery in Spain and Italy, there is collaboration with universities, research institutes and other stakeholders in some regions, which also affords access to other national and international funding support. In Switzerland and the Netherlands, the programmes are university-based with funding support from both the university and the government as well as research grants; both centres perform general WHS, collaborate with other agencies, institutes and stakeholders and take consulting and coordinating roles with regard to targeted surveillance and other wildlife health initiatives. In Great Britain, the current programme (GBWDSP) is delivered by a collaborative partnership between governmental agencies, non-governmental organisations and academia. Funding comes from both governmental and non-governmental sources.

Programme growth has been commonly driven by the need for increased targeted surveillance to minimise the impact on livestock and public health of pathogens found in wildlife and to meet reporting obligations by government agencies. However, specific events also fostered growth spurts of established programmes, and the awareness of such opportunities may be helpful when developing WHS schemes. The drafting and implementation of new plans and schemes, such as the GBWDSP in Great Britain and the PVSFS in Spain, were major events leading to significant growth spurts. In Spain, the development of an action plan on a specific disease (wildlife tuberculosis, PATUBES [20]) further stimulated the national PVSFS scheme. Organisational restructuring within the hosting university and the redefinition of mandates were fundamental events in the growth of the WHS programme in Switzerland. Events that significantly impacted the development of the DWHC included the detection of zoonotic pathogens in wildlife previously not known to occur in the Netherlands, the collaboration with a more experienced wildlife health organisation (The Canadian Wildlife Health Cooperative, http://www.cwhc-rcsf.ca/, accessed on 21 June 2021) and an external audit by a panel of international experts. Their positive evaluation provided assurance to the Dutch government that the DWHC was on the right track.

At the workshop, participants involved in established WHS programmes shared the following recommendations, based on actions that had worked for them. The coordination and harmonisation of pre-existing wildlife health efforts and collaboration with field networks, including the development of citizen science initiatives (e.g., https://www.gardenwildlifehealth.org/, accessed on 21 June 2021) facilitate resource-efficient programme development and delivery. Public outreach, the assignment of local contacts for stakeholders and the sharing of results are also important in ensuring optimal communication, the motivation of submitters and knowledge dissemination. Regular programme assessments and consultations with stakeholders, coupled with programme alignment with government priorities, facilitate adaptive programme development. Participation in international networks (e.g., African Swine Fever (ASF)-STOP Cost Action https://www.cost.eu/actions/CA15116/, accessed on 31 July 2021) can also boost programme growth. Further recommendations to add value to existing programmes include the establishment of sample and data archives, integration of WHS data with wildlife population data in order to help measure impacts of disease on biodiversity (e.g., [21]), cross-border coordination of WHS initiatives for transboundary species [22] and adoption of a One Health approach to programme delivery [23].

### 3.2. Challenges to WHS Programme Start Up and Development

During the workshop, several challenges to programme start up and expansion were identified both within established programmes and by countries setting up programmes. By far the largest challenge is a lack of funding or constraints and uncertainty regarding the continuity of existing funding. Additionally, infrastructure limitations and difficulties in maintaining a core of experienced personnel can limit programme capacity and expansion. Similarly, meeting differing obligations and expectations from various funders and partners (e.g., government versus academia) can be challenging. The political framework and degree of regional autonomy may complicate the transition from isolated, regional programmes to a harmonised, national programme. Distinct institutional frameworks for biodiversity and nature management versus disease control can also be a challenge to development. The lack of well-designed, centralised WHS databases with standardised data fields may also hamper start-up and coordination. Legal restrictions regarding necessary licences and permits (e.g., appropriate laboratory biosafety level, legislation-compliant transport systems for biological specimens) are another potential barrier. Limits in diagnostic testing due to unvalidated or expensive tests, and unequal representation of taxa and geographic regions, can negatively impact programme scope. Finally, the willingness and commitment of key actors involved in wildlife health initiatives to work together to reach a consensus on a common strategy is a pre-requisite for successful national programme development.

## 4. Discussion

During the workshop, participants discussed their experiences of how best to deal with the difficulties they had experienced in both established and starting WHS programmes. Their recommendations are formulated below, organised according to ten categories of challenges that were identified:

### 4.1. Challenge 1: Understanding and Awareness

*The general public and stakeholders often have limited understanding of the importance of wildlife diseases.*

Recommendations: Communicate the significance of wildlife diseases and the value of WHS, which can benefit public and domestic animal health, as well as wild animal health, welfare and conservation. Support this argument with international examples where anthropogenic activity (e.g., land-use change, habitat loss and trade) has been identified as a driver of disease emergence in, or pathogen spillover from, wildlife populations. Outline native and non-native wildlife species that may act as reservoirs of pathogens causing diseases of economic importance, with trade implications for livestock. Emphasise how emerging infectious and non-infectious diseases have caused significant wildlife population declines, local extirpations and even extinctions. Recognise growing societal recognition not only of the intrinsic value of biodiversity but also of nature engagement as a means to promote human health and well-being, which relies on healthy and resilient ecosystems. Capitalise on public concern for issues adversely impacting wildlife health and welfare and on high-profile media coverage that can result from it (e.g., following mass mortality incidents). Highlight how wildlife can serve as a sentinel of wider ecosystem health.

Be patient and inclusive, and tailor communication to meet the interest of varied stakeholder groups; pay attention to fostering good contacts with collaborating organisations who can help further the initiative by disseminating information and raising awareness amongst their colleagues and memberships.

### 4.2. Challenge 2: Cross-Sectoral Scope

*Activities are typically focused on public and/or domestic animal health whilst wildlife health and conservation are neglected.*

Recommendations: Promote the One Health approach (https://www.oie.int/en/for-the-media/onehealth/, accessed on 21 June 2021), highlighting the interconnection of the health of humans, wild and domestic animals and ecosystems. Encourage multidisciplinary initiatives that bring together wildlife, domestic animal and public health sectors, in partnership with social scientists and ecologists, to facilitate comprehensive WHS, and maximise the benefits of the information gained. Communicate to identify common interests and the benefits of collaborative working for each sector in order to ensure all have a clear incentive to participate. Ensure that risks to wildlife and ecosystem health are considered with particular attention, in addition to domestic animal and human health: participants in field reporting networks are often motivated by wild animal welfare and conservation concerns.

### 4.3. Challenge 3: National-Scale Collaboration

*Early stage WHS programmes often operate in isolation.*

Recommendations: Audit existing WHS activities, including programmes with taxon-specific remit, and focus on targeted threat detection or regional limitation. Perform a literature review and seek expert opinion to identify known endemic and emerging diseases, prioritise surveillance activities to incorporate within WHS schemes and conduct horizon scanning (i.e., systematic information scanning) for the early detection or prediction of future threats. Identify stakeholders from across the government, academia, non-governmental organisations on animal welfare and conservation, wildlife rehabilitators, hunters, farmers, land managers, etc. Review stakeholder goals and values and identify where these align or differ, since this may assist with understanding motivations to participate. Emphasise the benefits and synergies that can result from multi-disciplinary working amongst the wide range of parties with an interest in wildlife health [13]. Host workshops and/or conduct a scoping survey in order to identify shared priorities and encourage joint ownership and investment. Use these to develop a collaborative WHS strategy with clearly outlined aims, terms of reference and responsibilities. Establish regular networking opportunities at a national level (e.g., organise meetings, workshops and conferences, form partnerships and create a national wildlife health society). Recommend incremental change with the integration of existing schemes where possible in order to achieve shared goals.

### 4.4. Challenge 4: Harmonisation of Methods

*It is not possible to compare data across existing schemes that employ different methods.*

Recommendations: Explain the value of the harmonisation of methods to enable data sharing [e.g., EWDA Diagnosis Cards (https://ewda.org/diagnosis-cards/, accessed on 21 June 2021) and harmonised Approaches in monitoring wildlife Population Health, And Ecology and Abundance (APHAEA; https://www.aphaea.eu/, accessed on 21 June 2021)]. Develop set case and incident definitions and standard examination protocols. Adopt international schemes for sample archive collation for particular taxa (e.g., [24]). Establish memorandums of understanding and data-sharing agreements that are General Data Protection Regulation-compliant amongst collaborating organisations. Build a centralised national WHS database, perhaps developed on models from existing WHS programmes (e.g., Canadian Wildlife Health Cooperative’s Wildlife Health Intelligence Platform (http://www.cwhc-rcsf.ca/wildlife_disease_database.php, accessed on 21 June 2021)). Develop national reporting networks utilising online or mobile-based technologies (e.g., EpiCollect https://five.epicollect.net/, accessed on 21 June 2021). Consult available resources that outline best practice and provide guidelines relevant to WHS in order to optimise the approach and benefit from the lessons learned (e.g., [4,5,11]).

### 4.5. Challenge 5: Governmental Support

*Government funding is often insufficient, short-term and comes from one department (e.g., Agriculture or Public Health).*

Recommendations: Convince the government that WHS is an integral part of national disease surveillance and early threat detection, and that departments involved in the environment, agriculture and public health should co-fund WHS. This approach would foster efficient multi-disciplinary working and maximise cost-efficiency by avoiding silo working (i.e., independent work within isolated disciplines or departments), which risks a duplication of effort. Make programmes indispensable to the government as a source of information and expertise on wildlife health. Identify governmental and institutional obligations where WHS data may contribute (e.g., EU Open Data Portal https://data.europa.eu/euodp/en/data/, accessed on 21 June 2021). Explain how WHS can contribute data for government to meet their obligations under international treaties/legislation that focus on certain taxa (e.g., Agreement on the Conservation of Small Cetaceans in the Baltic, North Eastern Atlantic Irish and North Seas (ASCOBANS) https://www.ascobans.org/, accessed on 21 June 2021, and Convention on the Conservation of Migratory Species of Wild Animals (CMS) https://www.cms.int/, accessed on 21 June 2021) and the occurrence of diseases of international and national concern (e.g., OIE-Listed diseases https://www.oie.int/en/what-we-do/animal-health-and-welfare/animal-diseases/, accessed on 31 July 2021).

### 4.6. Challenge 6: Academic Support

*Academic recognition of the value of WHS is often lacking.*

Recommendations: Communicate with universities regarding how participation in a WHS programme provides an opportunity to educate students, undertake research and serve society, meaning that they should support and/or co-fund these initiatives. Illustrate with examples, e.g., the Canadian Wildlife Health Cooperative model (http://www.cwhc-rcsf.ca/, accessed on 21 June 2021), which utilises a coordinated network of diagnostic centres based at veterinary schools. Highlight the growing interest and demand amongst undergraduate and postgraduate student communities for wildlife health training opportunities (e.g., Wildlife Population Health residency programme of the European College of Zoological Medicine https://www.eczm.eu/wildlife/, accessed on 21 June 2021). Recognise the importance of research impact assessments for the performance appraisal of academic research institutes and how WHS can generate relevant findings for conservation, public and domestic animal health that can be translated into practical disease mitigation and management recommendations (e.g., biosecurity and habitat management). Share examples of successful grants attributed to wildlife health research.

### 4.7. Challenge 7: Other Funding Support

*It is challenging to secure funding.*

Recommendations: WHS programmes provide a valuable source of knowledge to inform the wildlife disease research priority setting, such as the early detection of novel and emerging threats. Long-term sample archives of national WHS programmes can be used to conduct retrospective targeted investigations at low cost. Develop integrated WHS and research programmes, which are mutually beneficial and represent a well-prioritised, coordinated and cost-effective approach that is attractive to governmental and scientific funders. Consider other funding sources (e.g., non-governmental organisations for animal welfare or conservation, private donors and charitable foundations) who may be interested in providing financial support for specific surveillance activities related to particular taxa, threats and/or elements that relate to public engagement, outreach and education.

### 4.8. Challenge 8: Staff Expertise and Capacity

*It is difficult to recruit, train and maintain expertise in staff.*

Recommendations: Explain to the government and university funders that it takes time to build up knowledge of wildlife health in a country, and that long-term secure funding is essential in order to train wildlife health experts and maintain institutional and national capacity. Encourage WHS programme staff participation in the wildlife health training of undergraduate and postgraduate students across a range of disciplines. Promote information sharing through national and international exchange networks (e.g., EWDA Network https://ewda.org/ewda-network/, accessed on 21 June 2021) and scientific conference presentations (e.g., Wildlife Disease Association https://www.wildlifedisease.org/wda/, accessed on 21 June 2021) in order to engage with the wider wildlife health community and help establish opportunities for staff skills and experience development.

### 4.9. Challenge 9: Leadership, Feedback and Engagement

*Problems often arise with maintaining momentum and keeping stakeholders engaged in the long term.*

Recommendations: Schemes at an early stage of development often rely on the efforts of a leader, or small leadership team, who instigates the programme, acts as a responsive regional contact point and devotes time to support relationship building amongst stakeholders. Develop a communication strategy that outlines the frequency and type of feedback to be provided to case reporters, field workers and stakeholders in order to meet expectations and thereby maintain engagement. Regularly disseminate outcomes and recommendations from the programme in formats accessible to the public (e.g., website, newsletter, open access peer-reviewed publications and social media). Establish specific people to act as regional contact points for stakeholders and promote both ‘bottom up’ and ‘top down’ feedback. Submit regular reports on listed and non-listed wildlife diseases to OIE through the World Animal Health Information Service (WAHIS)-Wild (https://www.oie.int/en/what-we-do/animal-health-and-welfare/disease-data-collection/world-animal-health-information-system/, accessed on 31 July 2021) and contribute to the annual reports of the OIE Working group on Wildlife, which are disseminated internationally via the National Wildlife Focal Points and are available from the OIE website (https://www.oie.int/en/what-we-do/standards/standards-setting-process/working-groups/working-group-on-wildlife/, accessed on 31 July 2021).

### 4.10. Challenge 10: Threat Mitigation and Wildlife Disease Management

*There is a poor translation of WHS findings into practical action or intervention.*

Recommendations: Encourage data collection on the distribution and density of wildlife in a harmonised way ([25]; e.g., ENETWILD; https://enetwild.com/, accessed on 21 June 2021) in order to allow for the quantification of the population scale impact of wildlife diseases and risk factor identification, which is crucial for wildlife population and disease management. Establish links to governmental and non-governmental organisations in relevant areas (e.g., animal health, public health, environment, conservation, hunting) to optimise knowledge transfer to management and policy. Utilise WHS data to inform disease risk assessments for conservation interventions, such as species translocation or the reintroduction [26] and development of non-native/invasive species management plans. Contribute WHS expertise in order to facilitate early warning and preparedness, and to develop contingency disease response plans where appropriate. Promote the open access publication of disease action plans and their application in order to facilitate the international adoption of successful and evidence-based approaches and to foster international cooperation to mitigate threats of transboundary diseases.

## 5. Conclusions

Diseases continue to emerge at the wildlife–domestic animal–human interface and there is an urgent need for the establishment of WHS programmes worldwide. The resource provided by the workshop may enable the further development of WHS in Europe and beyond.

## Figures and Tables

**Table 1:** Tentative compilation of the definitions of general and targeted surveillance. The following sources of information were taken into consideration [4,5,6,7].

	General/Scanning ^1^	Targeted/Hazard-Specific ^2^
Material collection ^3^ (often used to describe the type of surveillance)	**Passive** (=observer-initiated) **Cases received as they occur**, are found and submitted = opportunistic (whole carcasses or samples)	**Active** (= investigator-initiated) **Proactive sampling** or search of disease cases or information (mostly samples, e.g., blood, organs or whole carcasses) usually according to a **pre-defined** sampling plan: - Population size? - Prevalence estimation vs. freedom of disease? - Stratification: sex, age, geographical distribution, season, etc.
Objective	Searching for **any** **disease** within a population → detection of cases/signals (early warning; monitoring of disease trends)	Detect a **specific** **health hazard**: most often a pathogen, also e.g., a toxic compound or anomaly (early warning; demonstration of disease freedom and early detection of emerging pathogens; monitoring of endemic pathogens; success of control measures, etc.)
Health status of investigated animals	**Disease focus** (≈clinical surveillance: detecting dead or live but visibly sick animals)	Apparently healthy or diseased = **independent of health status**, whether alive or dead «Weighted surveillance»: when focused on a high-risk subset of the population (e.g., looking for a specific pathogen in all animals found dead or showing signs of illness)
Geographical area and animal species	Usually **all** that are covered by the health programme	Pre-defined, often **risk-based**
Population-level inference	Poor/**tip of iceberg**	**Improved—good** (depends on data collection strategy & usual limitations such as the access to wildlife samples)
Investigation method	**Pathology**, clinical exam, … Further tests as needed	Standardized, systematic procedure: Mostly **antigen detection** Others: histology, toxicology, serology, etc.
Classification according to (1)	**Level 1: no general surveillance** Absence of any programme of general WHS, but existence of limited targeted surveys of a few selected diseases **Level 2: partial general surveillance** Existence of a wide range of programmes including detection, diagnosis and management of disease-related information, but restricted in various ways (e.g., species spectrum, geographical range) **Level 3: comprehensive general surveillance** Existence of one or several programmes covering the entire country and being comprehensive with respect to species of animals examined and types of diseases investigated	

^1^ General/scanning surveillance is defined as the pathological or clinical examination of animals found dead or moribund, typically involving investigation for the diagnosis of a range of infectious and/or non-infectious diseases. ^2^ Targeted/hazard-specific surveillance is defined as the testing of animals for the presence of specific pathogens. ^3^ The type of surveillance is often called after the method of material collection (passive/active).
